# Editorial: Advances in techniques for measurement and assessment of physiological processes in developing animals

**DOI:** 10.3389/fphys.2023.1297691

**Published:** 2023-10-11

**Authors:** Huseyin C. Yalcin, Ahsan H. Khandoker, Koichi Kawakami

**Affiliations:** ^1^ Qatar University Biomedical Research Center, Doha, Qatar; ^2^ Department of Biomedical Engineering, Healthcare Engineering Innovation Center, Khalifa University, Abu Dhabi, United Arab Emirates; ^3^ National Institute of Genetics, Mishima, Japan

**Keywords:** zebrafish, chicken embryo, physiology, pig, honey bee, crab, embryo, heart

Developmental Physiology can be broadly defined as the field of science aiming to understand the physiological processes shaping a fertilized egg of a species into a well-structured and functional multi-cellular organism. Animal studies are a major part of such investigations, during which animal embryos and newly born or newly hatched animals are examined to understand complex developmental processes. At the organ level, these examinations include morphological assessments for structural analysis, and physiological assessments for functional analysis of developing organs/systems, whereas at cell/tissue level, relevant analyses are carried out with molecular biology and biochemistry assays. Precise measurement of physiological parameters is required for understanding developmental processes of specific organs and organ systems in such examinations. Even though, there is a growing interest from the Developmental Biology community for such experimental techniques, most of these applications have been developed for specialized research labs and are not readily available for other interested researchers. This Research Topic aimed to present novel experimental techniques on developing animals for adaptation of useful techniques by other researchers, advancing the field of Developmental Physiology.

Traditionally, mammalian models such as mice and rats have been the most commonly used and dominant animal models in developmental biological research, for obvious reasons. Yet, non-mammalian species also provide unique advantages in such investigations on developmental processes such as ease of culture, experimentation, and access to the embryos. In recent years, we have witnessed an increased interest and new application areas for a variety of non-mammalian developmental animal models. The Zebrafish, *Danio rerio,* has been a popular animal model in the field, particularly with the advantage of transparency at early stages enabling real time visualization of internal organs. As such, the Research Topic includes three zebrafish papers presenting useful experimental techniques for different applications. Messerschmidt et al. presents an elegant technique for DNA plasmid delivery to Zebrafish endocardium via poly (lactic-co-glycolic acid) (PLGA) nanoparticles injection. This nanomedicine approach was found to be non-toxic as well on zebrafish. Interfering with gene expression via plasmid delivery has been applied for the first time on zebrafish and is expected to be adapted in similar future studies with zebrafish. This study successfully demonstrated PLGA nanoparticle-mediated target delivery to upregulate Notch related genes would be a potential cardiac therapeutic approach with minimum toxic effects. The other two zebrafish papers in the Research Topic are relevant to assessment of cardiac function. Transparency of zebrafish embryos enable monitoring red blood cells (RBCs) for measuring blood flow velocities and calculation of wall shear stress acting on vessel walls. Real-time and continuous tracking of RBCs for this purpose is a significant challenge especially if multiple vessels are to be tracked simultaneously. Maung Ye et al. have established a semi-automated high-throughput fluorescent imaging system to capture the flow of RBCs in an entire zebrafish between 2- and 6-day post-fertilization (dpf) and successfully imaged as many as 50 embryos simultaneously. Similar to blood vessels, the zebrafish embryonic heart is accessible during embryonic stages for imaging and interference. This unique future enables studying whether the embryonic heart is capable of acute adaptation of electrical and mechanical activity in response to changes in mechanical load via feedback processes known as “mechano-electric coupling” and “mechano-mechanical coupling”. These processes are well defined for adult heart but are not well studied for developing hearts. Baillie et al. presents a novel approach for the *in vivo* study of mechano-electric and mechano-mechanical coupling in the developing zebrafish heart, where acute *in vivo* atrial dilation (i.e., increased atrial preload) in larval zebrafish is achieved by a controlled injection into the venous circulation, combined with optical measurement of the acute electrical (change in heart rate) and mechanical (change in stroke area) response.

The embryonic chicken is another popular animal model for developmental studies due to close resemblance of organ systems of the species with that of human, both morphologically and physiologically. One particular such system is the cardiovascular system. The avian heart develops very similar to human heart with a four chamber four valve configuration. Throughout the years, many surgical techniques has been developed on this model enabling interference with heart development to better understand the origins of congenital heart defects (CHDs) in human. Left atrial ligation, vitelline vessel clipping, and conotruncal banding are among the most popular techniques. In the Research Topic, Alser et al. introduced a new surgical approach called right atrial ligation (RAL) for inducing hemodynamic alterations in the right side of the heart and for examining its consequences. The authors concluded that RAL does not induce severe flow disturbance and ventricular abnormalities consistent with clinical findings, allowing a better understanding of the hemodynamics-driven CHD development. The avian digestive system has remarkable similarities with humans, making the chicken embryo model appropriate for such studies. Shikaya et al. present an LED light-stimulating apparatus for optogenetic control of gut movements in the chicken embryo. Gut peristalsis, recognized as a wave-like progression along the anterior-posterior gut axis, plays a pivotal role in the transportation, digestion, and absorption of ingested materials. The embryonic gut, which has not experienced ingested materials, undergoes peristalsis, offering a powerful model for studying the intrinsic mechanisms underlying gut motility. Induced artificial peristaltic waves enabled the authors to examine critical processes for gut peristalsis to explore therapeutic methodologies for peristaltic disorders.

In addition to studies on mammalian and non-mammalian established animal models, there is also a large number of developmental physiological studies on non-model wild, exotic, or farm animals. Monitoring physiological processes in such species is challenging and requires applying specialized tools and techniques. The Research Topic includes three articles in that category. Intrauterine growth restriction (IUGR) in humans often manifests as poor growth and delayed intellectual development, whereas in domestic animals, it increases mortality. As a novel epigenetic regulatory molecule, tRNA-derived small RNAs (tsRNAs) are reportedly involved in many biological processes. Gan et al. used pigs (35 days) as a model to characterize tsRNAs by sequencing in normal and IUGR porcine skeletal muscle, uncovering the role of tsRNAs in IUGR porcine skeletal muscle development, thus providing insights into the prevention and treatment of IUGR in mammals. Jiang et al. developed a new device to test the hypothesis of mutual attraction behavior between male and female crabs with mature gonads by releasing the sexual pheromone so they could be examined. A novel polygon shape device, looking like a maze, comprised a discriminating area, a transparent cover plate located at the top of the area, and an external mechanical aerating device. Using the device, authors successfully isolated Chinese mitten crabs with mature gonads hence successfully showed that, ovarian development in the experimental group was higher than in the control group and was nearly fully mature, confirming the initial hypothesis. Yan et al. developed an *in vitro* approach for investigating spermatophore development and its effect on metabolite support for sperm storage in honey bees, *Apis cerana*. Here, the authors compared the developmental processes and metabolites of seminal vesicles of drones from different parentages (0–24 days) in honeybee colonies, including mated queens, virgin queens, and worker bees. Notably, the metabolomics of the seminal vesicles in drones from mated queens showed different metabolites in each stage. Particularly, squalene identified among them was validated as a protective effect on sperm vitality for *in vitro* experiments.

Presenting unique experimental applications to study developmental physiological processes in developing animals, we believe this Research Topic will provide valuable insights for researchers using the animal models presented here.

